# IV.—Mithridatium

**Published:** 1895-04-13

**Authors:** 


### The Panacea in English Medicine.

(*Continued from page* 398, *Vol. XVII.*)

### IV.—MITHRIDATIUM.

If age can confer dignity, then the twin compositions known as theriaca and mithridatium must be raised to the first place among universal medicines. Their history, extending over a course of nearly two thousand years, embraces almost every variety of medical superstition and popular credulity, and nothing can more strongly illustrate the growth of modern science than the fact that a remedy able to show so unparalleled a record of unbroken success from the first century B.C. until well into the eighteenth century should have faded into an oblivion as complete as it is well deserved in the present day.

The first invention of the compound is ascribed to Mithridates, King of Pontus, who, living in daily dread of assassination by poison, devised an antidote to render him immune from its effects. Many receipts were handed about after his death for preparing the antidote to which he gave his name, but sceptics have averred that the only one found in his cabinet consisted of twenty leaves of rue, a grain of salt, two nuts, and two dried figs. However that may be, an elaborate preparation under the name of mithridatium, composed of 38 simples, soon became the vogue in Rome, and was the recognised safeguard of many Roman Emperors, who were wont to mix it with their own hands. Andromachus, the body physician of Nero, lent himself to improve upon it; he introduced a number of other ingredients, which brought up the total to 75, and by adding the flesh of vipers to the rest, greatly increased its efficacy in public esteem, and procured it the title of theriac (Greek, θηριον—viper).

In the Middle Ages disputes concerning the composition raged fiercely. It was just such a medicine as was peculiarly proper to serve the purpose of the charlatan or saltimbanco. The variety of the ingredients and the absolute impossibility of testing their origin led the way to romancing and imposture of unlimited extent. Not content with assigning to theriac the power of expelling poison from the system, and rendering the patient safe from after-doses, physicians united in the belief that it conveyed the power of resisting and rejecting all diseases. If it could be had genuine, few doubted but that it would prove a veritable panacea.

Accordingly, early in the seventeenth century all the best chemists set their wits to work at its reformation. The movement began in Rome and Bologna, and extended rapidly to Germany, France, and England. Du Chesne, or, as he preferred to call himself, Quercetanus, devoted himself to harmonising all the ancient recipes, and the work was finally achieved in France to everybody's satisfaction by M. Daquin, the leading physician to Louis XIV.

Desperate offence was given to the English apothecaries by the pretension early set up on the Continent that this famous drug could never be rightly prepared in England, for want of the best ingredients. A curious little black letter tract by R. Brown, bearing date 1612, on the efficacy of "London Tryacle," puts the case for the English apothecary. Browne complains that "a filthy and unwholesome baggage composition termed commonly Triacle of Geane, hath been craftily, in a monstrous quantity, some thousand weight yearly, brought into this realm . . . being made only of the rotten garble and refuse outcast of all kinds of spices and drugs, hand over head, with a little filthy molasses and tar to work it up withal." This "false and naughty kind of mithridatium," as it is elsewhere called, was sold at 3d. a pound, very much, it may be believed, to the detriment of the public, until the masters and wardens of the Grocers' Company undertook the manufacture of the antidote under the direct authority of the Lord Mayor and Corporation of the City of London.

A discourse delivered at Nancy, 1725, by Charles Bagard, physician to the Dauphin, explains very succinctly wherein the extreme rarity of the right article consisted.

In the first place, much depended upon getting the right kind of viper. "It must issue living from the womb of its mother and not in eggs as do other species"; it must be chosen fat and vigourous in a good season, the head and tail cut off, and the trunk, liver, and heart dried and pounded. This was the great antidote to poison. Castoreum, obtained from the beaver, was extremely dear and difficult to obtain, and was held to have great value in the medicine. But the most precious of all the ingredients was the opobalsamum, balm of Mecca, or true balm; it was taken from a tree, given by the Queen of Sheba to Solomon, which flourished only in Jericho. From there it was transplanted by the Turkish Emperor to Cairo, where it was so carefully guarded, that only by favour of the Turkish ambassadors could any penetrate to Europe. Its extreme rarity enhanced its value, and no theriaca was worth having without it. Bitumen from the Dead Sea was a valued ingredient, from its virtue "in resisting malignity" and opium in somewhat large quantities, taken from the black poppies of Egypt and Greece, was by far the most active member in this heterogeneous partnership of drugs. The juices and gums were dissolved in good Spanish wine, and the remaining ingredients were pounded together with honey. The mixture was left to ferment for some weeks. At the end of six months it was fit for use, and if properly prepared, was thought to retain its efficacy for forty or fifty years.

It is needless to recall testimonies to the reputation of mithridatium. Even before its revival in Europe in the sixteenth century, no fewer than 1,500 books had been written on its virtues. Bagard testifies, "if one may believe all that authors have written, no other remedies are necessary. Theriac is a universal panacea, that is, a medicament proper for the cure of all the maladies to which the nature of man is subject."

The perfection to which the preparation of the drug was at length wrought, worked its downfall. When it was no longer possible to assign its failures to spurious ingredients there was no more hope for it. Dr. Heberden, writing in 1745, says, "it still goes on to be prepared in the old manner in all the great cities of Europe;" but he notes with satisfaction that "its power and fame has of late been manifestly declining, and we may hope its reign will not last much longer. Perhaps the glory of its first expulsion from a public Dispensatory was reserved to these times, and to the English nation." From this protest to the complete extinction of the drug, the time was short.

